# Detection of diploid males in a natural colony of the cleptobiotic bee *Lestrimelitta* sp (Hymenoptera, Apidae)

**DOI:** 10.1590/S1415-47572010000300019

**Published:** 2010-09-01

**Authors:** Mara Garcia Tavares, Carlos Roberto Carvalho, Fernanda Aparecida Ferrari Soares, Anderson Fernandes

**Affiliations:** 1 Departamento de Biologia Geral, Universidade Federal de Viçosa, Viçosa, MGBrazil; 2 Departamento de Ciências Biológicas, Universidade do Estado de Mato Grosso, Mato Grosso, MTBrazil

**Keywords:** cytogenetic, flow cytometry, genome size, karyotype, stingless bee

## Abstract

When working at quantifying the genome size of stingless bees, it was observed that males of *Lestrimelitta* sp possessed the same amount of nuclear DNA as the females. Thus, we used flow cytometry (FCM) and cytogenetic analysis to confirm the ploidy of these individuals. The males analyzed proved to be diploid, since, through cytometric analysis, it was demonstrated that the mean genome size of both males and females was the same (C = 0.463 pg), and, furthermore, cytogenetic analysis demonstrated that both had 2n = 28 chromosomes.

In Hymenoptera, sex is determined by haplodiploidy and in several species it is regulated by a single, multi-allelic sex locus (sl-CSD) (Beye *et al.*, 2003). In this model, hemizygous individuals will become males (haploid drones), whereas diploid individuals that are heterozygous at the sex locus will develop into females and those homozygous into diploid males.

In general, diploid males are less viable (Whiting, 1943; Rothenbuhler *et al.*, 1968), and either sterile (El Agoze *et al.*, 1994; Duchateau and Marien, 1995; Krieger *et al.*, 1999) or produce diploid sperm which leads to triploid offspring (Naito and Susuki, 1991; Liebert *et al.*, 2005; de Boer *et al.*, 2007), thereby imposing a genetic load on the population as a whole. In only one of the species studied so far, *Euodynerus foraminatus,* fertility is normal in diploid males, with the production of diploid, rather than triploid female offspring (Cowan and Stahlhut, 2004).

Diploid males have been detected in more than 60 species of Hymenoptera, this including several species of bees (both social and solitary), wasps, ants, sawflies and parasitoids. Among the stingless bees, they have been observed only in *Melipona compressipes, M. quadrifasciata, Scaptotrigona postica, Trigona carbonaria* and *Tetragona quadrangula* (van Wilgenburg *et al.*, 2006, Heimpel and de Boer, 2008), but not so in the genus *Lestrimelitta*.

*Lestrimelitta* is an essentially cleptobiotic (robber) stingless bee that exploits the resources of other bees by stealing food from their nests, instead of collecting it from flowers (Sakagami and Laroca, 1963; Bego *et al.*, 1991; Sakagami *et al.*, 1993). The genus occurs in the Neotropical region (Michener, 2000), and is represented in Brazil by at least fourteen species (Marchi and Melo, 2006).

When dealing with the quantification of genome size in stingless bees, it was noted that, in a colony of *Lestrimelitta* sp obtained in Domingos Martins/Espírito Santo (20°21'48” S; 40°39'33” W), nuclear DNA content proved to be the same in both males and females. As diploid males had not been previously noted in this species, individual ploidy was thereupon confirmed by flow cytometry (FCM) and cytogenetic analysis.

For the FCM analysis, the nuclear DNA content of *Lestrimelitta* sp male and female larvae was measured by using the C DNA content (0.42 pg) of *Scaptotrigona xantotricha* as internal standard, as described by Lopes *et al.* (2009). Brain ganglion nuclei of the standard and sample were excised in physiological saline solution (0.155 mM NaCl). The material was simultaneously crushed 10 times with a pestle in a tissue grinder (Kontes Glass Company^®^) with 100 μL of OTTO-I lysis buffer (Otto, 1990) containing 0.1 M citric acid (Merck), 0.5% Tween 20 (Merck) and 50 μg mL^-1^ of RNAse (Sigma-Aldrich), pH 2.3. The suspension was adjusted to 1.0 mL with the same buffer, filtered through a 30 μm nylon mesh (Partec) and centrifuged at 100 *g* in microcentrifuge tubes for 5 min.

The pellet was then incubated for 10 min in 100 μL of OTTO-I lysis buffer, and stained with 1.5 mL of OTTO-I:OTTO-II (1:2) solution (30 min) (Loureiro *et al.*, 2006), supplemented with 75 μM of propidium iodide (PI) and 50 μg mL^-1^ of RNAse, pH = 7.8. The nuclear suspension was filtered through a 20 μm diameter mesh nylon filter and maintained in the dark for 5-40 min.

Three independent replicates of each suspension were analyzed with a Partec PAS flow cytometer (Partec) equipped with a Laser source (488 nm).

The mean genome size (pg) of each bee sample was measured according to a formula adapted from Dolezel and Bartos (2005).

For cytogenetic analysis, metaphase chromosomes were obtained from cerebral ganglia and testes of male larvae, and from cerebral ganglia of females in the final defecation stage (Imai *et al.*, 1988). On an average, 5 females and 15 males, with ten metaphases per individual, were analyzed. Conventional Giemsa staining, using a 0.06 M Sörensen buffer, pH 6.8, was carried out according to Rocha and Pompolo (1998).

A 12-bit CoolSNAP-Pro cf (Roper Scientific) video camera, assembled on an OlympusTM BX-60 microscope with a 100x objective, was used for capturing chromosome images. The frame was digitized using an Image Pro-Plus analysis system (Media Cybernetics). A Power Macintosh G4 computer was employed for image analysis, with freely available Image SXM software (Barrett, 2002). This is a spin-off of the public domain image analysis application NIH Image which was developed by Rasband (1998). The karyotype was mounted by pairing chromosomes in the order of decreasing size.

Cytometry analysis of nuclei suspensions stained with PI demonstrated that the mean genome size of both males and females was the same (C = 0.463 pg) (Table 1 and Figures 1a and 1c), thereby indicating that the males were diploid.

Cytological analysis confirmed female and male chromosome content to be 2n = 28 (Figures 1b and 1d), as already described for *Lestrimelitta limao* females (Rocha *et al.*, 2003). Neither cytometry nor cytogenetic analysis revealed haploid males among those analyzed.

The presence of diploid males is likely to generate high fitness costs for individual colonies and their queens, since there is a potential reduction in the proportion of workers performing essential tasks for colony survival (Green and Oldroyd, 2002). In colonies of stingless bees, workers construct and mass provision the cells prior to ovipositing. Thereafter, the queen lays her eggs in the cells, which the workers then seal (Sakagami, 1982). This prevents the detection and early removal of diploid males. In fact, it was noted that diploid males of *Lestrimelitta* sp presented normal viability in the larval and pupa phases, and fully developed into imagos. Nevertheless, several workers were seen attacking young diploid males inside the colony. Furthermore, the colony was weak, presenting several brood cells with dead progeny, with numerous mites attacking the larvae. This colony perished only a few days after being opened in the laboratory. Likewise, in colonies of *Bombusatratus* (Plowright and Pallet, 1979) and *Solenopsisinvicta* (Ross and Fletcher, 1986), the production of diploid males also retarded colony growth, with consequential high mortality.

Diploid male production has been attributed to habitat fragmentation, the loss of sex allele diversity by drift in small, isolated populations, and the mating of parents sharing a sex allele in common, *i.e.*, matched matings (revision in Cowan and Stahlhut, 2004). For *Lestrimelitta* in particular, the active human destruction of its colonies, in order to countering pillage of other stingless bee colonies, could have reduced species population size, thus favoring inbreeding, with diploid males as the possible outcome of matched mating. Consequently, since *Lestrimellita* females mate with a single male (Peters *et al.*, 1999), it may be inferred that sex in *Lestrimellita* is controlled by a single multiple-allelic locus.

**Figure 1:**
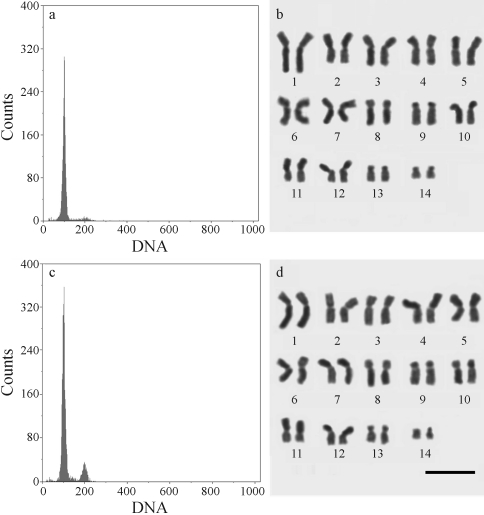
Genome size DNA-histograms and karyotype (Giemsa staining) of female (a and b) and diploid male (c and d) of *Lestrimelitta* sp. Bar = 5 μm.

## Figures and Tables

**Table 1:** Estimation of genome size of cerebral ganglia of *Lestrimelitta* sp.

	Mean genome size (1C; pg)			
	*R1	*R2	*R3	Mean ± SD
*Lestrimellita* sp (diploid males)	0.460	0.465	0.465	0.463 ± 0.003
*Lestrimellita* sp (females)	0.455	0.465	0.470	0.463 ± 0.008

*R1, *R2 and *R3 : independent replicates.

## References

[Beyeetal2003] Beye M., Hasselmann M., Fondrk M.K., Page R.E., Omholt S.W. (2003). The gene *csd* is the primary signal for sexual development in the honeybee and encodes an SR-type protein. Cell.

[Barrett2002] Barrett S.D. (2002). Software for scanning microscopy. Proc R Microsc Soc.

[Begoetal1991] Bego L.R., Zucchi R., Mateus S. (1991). Notas sobre a estratégia alimentar de *Lestrimelitta limao* Smith (Hymenoptera, Apidae, Meliponinae). Naturalia.

[CowanandStahlhut2004] Cowan D.P., Stahlhut J.K. (2004). Functionally reproductive diploid and haploid males in an inbreeding hymenopteran with complementary sex determination. Proc Natl Acad Sci USA.

[deBoeretal2007] de Boer J.G., Ode P.J., Vet L.E.M., Whitfield J., Heimpel G.E. (2007). Diploid males sire triploid daughters and sons in the parasitoid wasp *Cotesia vestalis*. Heredity.

[DolezelandBartos2005] Dolezel J., Bartos J. (2005). Plant DNA flow cytometry and estimation of nuclear genome size. Ann Bot.

[DuchateauandMarien1995] Duchateau M.J., Marien J. (1995). Sexual biology of haploid and diploid males in the bumble bee *Bombus terrestris*. Insectes Soc.

[ElAgozeetal1994] El Agoze M., Drezen J.M., Renault S., Periquet G. (1994). Analysis of the reproductive potential of diploid males in the wasp *Diadromus pulchellus* (Hymenoptera, Ichneumonidae). Bull Entomol Res.

[GreenandOldroyd2002] Green C.L., Oldroyd B.P. (2002). Queen mating frequency and maternity of males in the stingless bee *Trigona carbonaria* Smith. Insectes Soc.

[HeimpelanddeBoer2008] Heimpel G.E., de Boer J.G. (2008). Sex determination in the Hymenoptera. Annu Rev Entomol.

[Imaietal1988] Imai H.T., Taylor R.W., Crosland M.W.J., Crozier R.H. (1988). Modes of spontaneous evolution in ants with reference to the minimum interaction hypothesis. Jpn J Genet.

[Kriegeretal1999] Krieger M.J.B., Ross K.G., Chang C.W.Y., Keller L. (1999). Frequency and origin of triploid in the fire ant *Solenopsis invicta*. Heredity.

[Liebertetal2005] Liebert A.E., Sumana A., Starks P.T. (2005). Diploid males and their triploid offspring in the paper wasp *Polistes dominulus*. Biol Lett.

[Lopesetal2009] Lopes D.M., Carvalho C.R., Clarindo W.R., Praça M.M., Tavares M.G. (2009). Genome size estimation of three stingless bee species (Hymenoptera, Meliponinae) by flow cytometry. Apidologie.

[Loureiroetal2006] Loureiro J., Rodriguez E., Dolezel J., Santos C. (2006). Comparison of four nuclear isolation buffers for plant DNA flow cytometry. Ann Bot.

[MarchiandMelo2006] Marchi P., Melo G.A.R. (2006). Revisão taxonômica das espécies brasileiras de abelhas do gênero *Lestrimelitta* Friese (Hymenoptera, Apidae, Meliponina). Rev Bras Entomol.

[Michener2000] Michener C.D. (2000). The Bees of the World.

[NaitoandSusuki1991] Naito T., Susuki H. (1991). Sex determination in the sawfly, *Athalia rosae ruficornis*: Occurrence of triploid males. J Hered.

[Otto1990] Otto F.J., Darzynkiewiez Z., Crissman H.A., Robinson J.P. (1990). DAPI staining of fixed cells for high-resolution flow cytometry of nuclear DNA. Methods in Cell Biology.

[Petersetal1999] Peters J., Queller D.C., Imperatriz-Fonseca V.L., Roubik D.W., Strassmann J.E. (1999). Mate number, kin selection and social conflicts in stingless bees and honeybees. Proc R Soc Lond B.

[PlowrightandPallet1979] Plowright R.C., Pallet M.J. (1979). Worker-male conflict and inbreeding in bumble bees (Hymenoptera, Apidae). Can Entomol.

[RochaandPompolo1998] Rocha M.P., Pompolo S.G. (1998). Karyotypes and heterochromatin variation in *Melipona* species (Hymenoptera, Apidae, Meliponinae). Genet Mol Biol.

[Rochaetal2003] Rocha M.P., Pompolo S.G., Campos L.A.O., Melo G.A.R., Santos I.A. (2003). Citogenética da tribo Meliponini (Hymenoptera, Apidae). Homenagem aos 90 Anos de Jesus Santiago Moure.

[Rothenbuhleretal1968] Rothenbuhler W.C., Kulincevic J.M., Kerr W.E. (1968). Bee genetics. Annu Rev Genet.

[RossandFletcher1986] Ross K.G., Fletcher D.J.C. (1986). Diploid male production - A significant colony mortality factor in the fire ant *Solenopsis invicta* (Hymenoptera, Formicidae). Behav Ecol Sociobiol.

[Sakagami1982] Sakagami S.F., Hermann H.R. (1982). Stingless bees.

[SakagamiandLaroca1963] Sakagami S.F., Laroca S. (1963). Additional observations on the habits of the cleptobiotic stingless bees, the genus *Lestrimelitta* Friese (Hymenoptera, Apoidea). J Fac Sci Hokaido University Ser VI Zool.

[Sakagamietal1993] Sakagami S.F., Roubik D.W., Zucchi R. (1993). Ethology of the robber stingless bee *Lestrimelitta limao* (Hymenoptera, Apidae). Sociobiology.

[vanWilgenburgetal2006] van Wilgenburg E., Driessen G., Beukeboom L.W. (2006). Single locus complementary sex determination in Hymenoptera: An “unintelligent” design. Front Zool.

[Whiting1943] Whiting P.W. (1943). Multiple alleles in complementary sex determination of *Habrobracon*. Genetics.

